# Effectiveness of auditory distraction on the management of dental anxiety in patients undergoing tooth extraction at a tertiary care hospital in Islamabad

**DOI:** 10.1002/cre2.863

**Published:** 2024-03-03

**Authors:** Sheze Haroon Qazi, Saba Masoud, Khadija Tariq, Minahil Khan, Reza Emrani, Javed Ashraf

**Affiliations:** ^1^ Islamabad Medical and Dental College Islamabad Pakistan; ^2^ School of Dentistry Qazvin University of Medical Science Qazvin Iran; ^3^ Institute of Dentistry University of Eastern Finland Kuopio Finland; ^4^ Health Services Academy Islamabad Pakistan

**Keywords:** dental anxiety, randomized control trial, tooth extraction

## Abstract

**Objectives:**

The study aimed to assess the effectiveness of anxiety reduction protocol using auditory distraction in alleviating dental anxiety among patients undergoing tooth extraction.

**Materials and Methods:**

A randomized controlled trial was conducted at the Oral Surgery Department at Islamabad Dental Hospital from July to December 2022, involving 50 patients scheduled for tooth extraction. Participants were randomly divided into two groups: an interventional group, exposed to auditory distraction, and a noninterventional group, without exposure to auditory distraction before the dental extraction. Dental anxiety was measured using the modified dental anxiety scale (MDAS) questionnaire, which scores anxiety levels on a range from 5 (not anxious) to 25 (extremely anxious). Anxiety levels were assessed in the waiting room and just before extraction, and the results were compared across both groups to evaluate the effectiveness of auditory distraction in reducing dental anxiety.

**Results:**

The sample size of 50 was randomly and equally allocated to the interventional and noninterventional groups. The study population consisted of 28 (56%) female and 22 (44%) male participants. No significant difference was observed between the anxiety scores of interventional and noninterventional groups at baseline. A significant reduction in anxiety scores was observed in the intervention group during postintervention assessment, while no significant difference was seen in the noninterventional group's anxiety scores.

**Conclusions:**

The study supports the efficacy of anxiety reduction protocol using auditory distraction as a practical tool for reducing dental anxiety among patients undergoing tooth extraction.

## 1 INTRODUCTION

Dental fear and anxiety significantly contribute to the avoidance of dental care (Murad et al., 2020). Anxiety, characterized as a sequence of behaviors, involves psychophysical activation in response to unpleasant stimuli (Aliabadi et al., 2020). These stimuli can be either internal or external. Internal stimuli may be somatic and cognitive, whereby the patient is encouraged to think about something unrelated to the dental situation. Conversely, external stimuli comprise environmental factors that the patient experiences before, during, and after the procedure. The level of dental anxiety is associated with an increased perception of anticipated pain. However, this vicious cycle of pain and anxiety may be mitigated by providing patients with reassuring and informative communication (Perković et al., 2014). Dental anxiety is most commonly associated with post‐traumatic treatment experiences, detrimentally affecting the oral health‐related quality of life for both children and adults (Xu & Xia, 2020). The prevalence of dental anxiety in children and adults ranges from 5.7% to 20.2% (Tiwari et al., 2021). A study conducted among university students in Rawalpindi, Islamabad, and Multan in Pakistan revealed high to severe dental anxiety in 1.6% of males and 24% of females (Shaikh & Kamal, 2011). Females, naturally inclined to avoid perceived threats both socially and biologically, report higher and statistically significant dental anxiety scores. Additionally, their exposure to distinct environmental and genetic factors, compared to men, may contribute to more pronounced responses toward phobias (Oosterink et al., 2009). Since anxiety can be triggered by the most incongruous situations, such as an encounter with the receptionist or the clinical ambiance, it is imperative to ensure that every aspect of the dental visit is optimized to alleviate dental anxiety (Appukuttan, 2016).

Corah's dental anxiety scale, the modified dental anxiety scale (MDAS), and the dental fear survey are the most commonly utilized scales for assessing dental anxiety (Masoud et al., 2022). Patients afflicted with dental anxiety tend to defer dental treatment and frequently exhibit nervous behaviors during dental visits, potentially straining the relationship between the dentist and the patient (Erten et al., 2006).

Dental anxiety can be managed using psychotherapeutic and pharmacological interventions. Psychotherapeutic interventions encompass behavioral and cognitive therapies, while pharmacological management involves the use of sedation and anesthesia. It is speculated that stress reduction may be achieved through relaxation and reassurance (Yoo et al., 2008).

As early as 1981, the impact of music as a form of distraction was investigated for its effects on pain among gynecologic/obstetric patients (Alhazmi et al., 2022). However, currently, there is a gap in the systematic approach to managing dental anxiety utilizing auditory distraction and catering factors such as optimal timing and duration. This study aims to address the effectiveness of dental anxiety‐reducing protocol using auditory distraction specifically before tooth extraction thereby providing a simple technique for dental practitioners to incorporate into their practice and improve the patient's experience.

## 2 METHODOLOGY

A randomized control trial was conducted from July to December 2022 with 50 patients at the Oral and Maxillofacial Surgery (OMFS) Department of Islamabad Dental Hospital (IDH). The study received ethical approval from the Institutional Review Board of IDH (Ref# IMDC/DS/IRB/231 Dated 5th July 2022). Only the patients aged 15−59 years, presenting for simple extractions (non‐restorable nonmobile teeth), were considered for inclusion in this study. Wisdom tooth extractions of any kind were excluded from this study. The randomization process involved systematic random sampling–every second patient visiting the OMFS department for simple extraction was selected. These patients were then alternatively assigned to noninterventional and intervention groups. The recruitment of these patients was spread over the entire study period, from July to December 2022. This approach ensured a staggered but systematic enrollment of patients, aligning with the flow of regular patient visits to the department.

**Figure 1:**
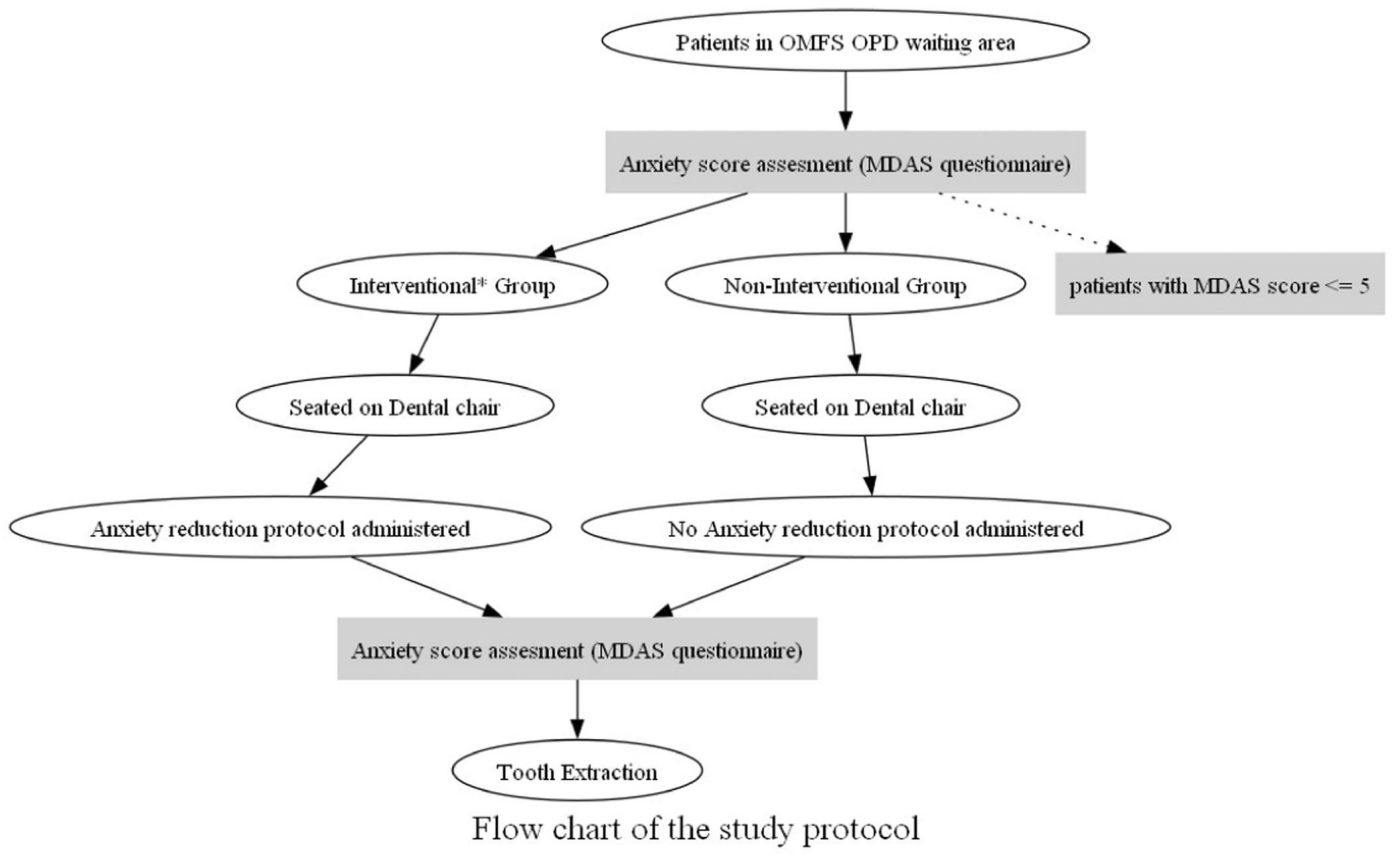
Flowchart describing the study's protocol. *The interventional group refers to participants receiving a combined protocol of anxiety reduction and auditory distraction. MDAS, modified dental anxiety scale.

Inclusion criteria included those who gave written informed consent and were categorized as dentally anxious (as assessed through the MDAS questionnaire with a total score exceeding 5). Also, patients with a history of mental illness, known allergy to local anesthetic, scheduled for surgical extraction, or language barriers were excluded from the study. It was hypothesized that there would be a difference in the mean anxiety scores in the intervention group as compared to the noninterventional group. The above‐mentioned procedure is visually summarized as a flow chart in Figure 1.

The English version of the MDAS questionnaire (Humphris et al., 1995), provided in Annexure 1, was translated into Urdu. Subsequent revisions were made to this translation, resulting in Cronbach's *⍺* value of 0.84. Section I of the MDAS questionnaire comprised questions on demographic variables such as age, gender, marital status, employment status, educational level, and past dental visits. Section II contained five questions about dental anxiety, scored on a Likert scale ranging from “not anxious (score 1),” “slightly anxious (score 2),” “fairly anxious (score 3),” “very anxious (score 4),” to “extremely anxious (score 5).” The total score, calculated as the sum of all five questions in Section II, provided a range of 5−25. Based on these total scores, categories were defined as “Not anxious (5),” “Mild anxiety (6−10),” “Moderate anxiety (11−14),” “High anxiety (15−18),” or “Extreme anxiety/dental phobia (19−25)”. After obtaining patient consent, the MDAS questionnaire was distributed in the waiting area. Patients were numbered and alternatively assigned to interventional and noninterventional groups.

The noninterventional group received no intervention, that is, auditory distraction, and the tooth extraction was performed routinely (anxiety reduction protocol steps 1−4) by the surgeon. For the interventional group, anxiety reduction protocol including audio distraction was given (steps 1−5), while the patient was in the dental chair immediately before tooth extraction. Patients from the interventional group were invited to select their preferred music composition or religious recitation and were provided with sanitized ear pods. The selected audio was played using a mobile phone for 5−10 min. To address potential confounders related to interoperator variability, all extractions were performed by the same senior postgraduate resident of the OMFS Department. The anxiety reduction protocol using audio distraction was provided by the primary researcher which included the following sequential steps:

1) Greeting the patient warmly on arrival.
2) Carefully listening to their concerns.
3) Verbal reassurance to the patient.
4) Giving the patient a way to communicate (raise hand if in pain or discomfort).
5) Audio distraction.

In both the interventional and noninterventional groups, patients' levels of anxiety were initially assessed in the waiting room, followed by a second evaluation immediately before the extraction while the patient was seated in the dental chair. This protocol was predicated on the hypothesis that anxiety would either peak or remain constant at this juncture, and then decrease post‐extraction.

For assessing the normality of continuous variables, the Shapiro−Wilk test was utilized. Consequently, a nonparametric test, the Wilcoxon signed‐ranks test, was utilized to examine the change in questionnaire scores (mean anxiety scores). The Mann−Whitney *U* test was applied to evaluate the distribution of mean anxiety scores across the interventional and noninterventional groups. Multivariable analyses of variance were used to assess the impact of various predictor variables on the mean‐anxiety scores of both groups, aiming to identify potential factors that may contribute to anxiety related to dental procedures.

A checklist from the Critical appraisal skill program (CASP) was used to check the strengths and weaknesses of this study (CASP [internet], 2023). The appraisal of the CASP list for this study reveals notable adherence to key research guidelines, in methodological rigor, and ensuring initial group equivalence. While there are areas for improvement, such as more detailed reporting on intervention effects and drop‐out rates, the study's foundational strengths offer a promising basis for future research advancements. This list is provided as Annexure 2.

The Statistical Package for Social Sciences (SPSS) version 22 was used for descriptive data analysis. Visualizations were made through Matplotlib and Seaborn packages of Python (version 3.11.5), whereas the flow chart was made through the Graphviz package of Python.

## 3 RESULTS

The study sample comprised 50 participants, split evenly across interventional and noninterventional groups (*n* = 25 per group), with a gender distribution of 28 females (56%) and 22 males (44%) and a mean age of 32.80 (SD = 10.33) years. The study sample characteristics are presented in Table 1.

**Table 1:** Sample characteristics of interventional and noninterventional groups.

Variables	Interventional group	Control group	*p* Value
Age,^a^(mean ± SD)	33.28 (11.85)	32.32 (8.77)	0.861^b^
Total pre‐anxiety score,^a^ mean (SD)	10.84 (3.47)	11.28 (3.40)	0.653^c^
Total post‐anxiety score,^a^ mean (SD)	8.96 (3.03)	11.12 (3.15)	0.014^b^
Gender, *n* (%)^a^			1.000^d^
Male	11 (50)	11 (50)	
Female	14 (50)	14 (50)	
Marital status, *n* (%)^a^			
Married	17 (53)	15 (47)	0.768^d^
Unmarried	8	10	
Education, *n* (%)^a^			
Yes	25	22	0.235^e^
No	0	3	
Employment status, *n* (%)^a^			1.000^d^
Employed	15 (50)	15 (50)	
Unemployed	10 (50)	10 (50)	
Previous dental history, *n* (%)^a^			1.000^d^
Yes	14	13	
No	11	12	

^a^ Mean ± SD for continuous variables, counts, and row‐wise proportions for categorical variables.

^b^ *p* Value based on Mann−Whitney *U* test.

^c^ *p* Value based on *t*‐test.

^d^ *p* Value based on *χ* ^2^.

^e^ *p* Value based on fishers' exact.

**Figure 2:**
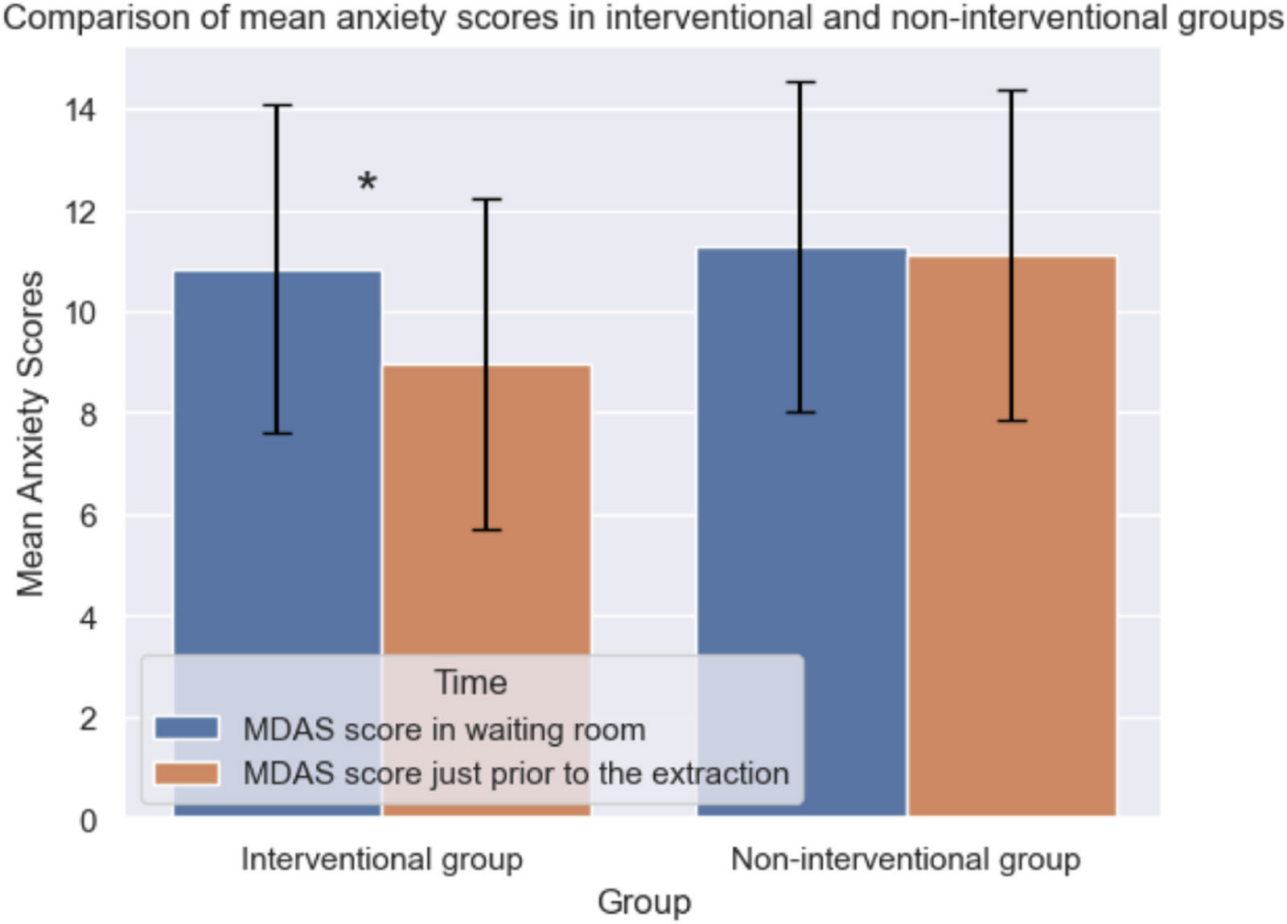
Comparison of mean anxiety scores in interventional and noninterventional groups. Error bars denote the standard deviation. *p < 0.05. MDAS, modified dental anxiety scale.

The mean anxiety score for both interventional and noninterventional groups was measured at two points, that is, baseline (in the waiting room) and before extraction (on the dental unit). The two groups were comparable at the baseline and there existed no significant difference in their anxiety levels as shown by the Mann−Whitney test (*p* = 0.592). However, the two groups showed a significant difference between their scores just before the tooth extraction (*p* = 0.013) (Table 2). Following the application of auditory distraction in the intervention group, the mean anxiety score significantly decreased. Conversely, in the noninterventional group, no significant difference was observed between the dental anxiety scores assessed in the waiting room and just before the extraction (*p* > 0.05) (Figure 2).

**Table 2:** Comparison of anxiety levels in the two groups at baseline and preprocedure.

Stage of observation	Groups	*N*	Mean rank	Range	*p* Value
Baseline (waiting room)	Intervention	25	24	5−20	0.592
	Nonintervention	25	26	5−17	
Preprocedure	Intervention	25	20.42	6−19	0.013^a^
	Nonintervention	25	30.58	6−18	

^a^ Significant *p*‐value according to Mann−Whitney test.

The multivariate analysis of variance model (Table 3) was utilized to investigate the effects of several predictors on two dependent variables, mean anxiety scores at baseline and just before extraction, in both interventional and noninterventional groups. The predictors included age, gender, marital status, education, employment status, dental history, and Intervention. The intervention variable had two categories: intervention and no intervention, with nonintervention being the reference category. According to this model, age, gender, marital status, education, employment status, and dental history were not significantly associated with the dependent variables (*p* > 0.05). However, the intervention variable was significantly associated with the dependent variables (*p* < 0.05). The rationale behind testing the relationship between categories of predictor variables and the mean anxiety scores was to identify potential factors that may contribute to mean anxiety scores related to dental procedures.

**Table 3:** Multivariate analysis of variance (MANOVA) based on pre and post‐anxiety scores.

Term/variable	Wilks' lambda	*F* Value	*p* Value
Intercept	0.6929	9.0842	0.0005
Age	0.9351	1.4236	0.2525
Gender^a^ (males)	0.9767	0.4899	0.6162
Marital status^b^ (unmarried)	0.9999	0.0016	0.9984
Education^c^ (educated)	0.9517	1.0393	0.3628
Employment status^d^ (unemployed)	0.9537	0.9951	0.3784
Dental history^e^ (no dental history)	0.9671	0.6971	0.5038
Intervention^f^ (interventional group)	0.5864	14.4592	0.0000

*Note*: The degree of freedom for the denominator is 41 and for the numerator, it is 2.

^a^ Reference category is “females.”

^b^ Reference category is “married.”

^c^ Reference category is “not educated.”

^d^ Reference category is “employed.”

^e^ Reference category is “positive dental history.”

^f^ Reference category is the “noninterventional group.”

The comparison of mean anxiety scores across intervention and nonintervention categories of the intervention variable before and after the intervention provided insight into the baseline differences and the effects of the intervention on the mean anxiety scores. As shown in Figure 2, a statistically significant (*p* < 0.05) reduction in the mean anxiety scores was observed in the intervention group following the application of auditory distraction.

## 4 DISCUSSION

This study's findings demonstrate that auditory distraction effectively reduces dental anxiety in patients undergoing nonsurgical extractions. Also, this study reveals that gender, marital status, education, employment status, and dental history do not exhibit any significant associations with anxiety levels.

The results of this study align with a randomized controlled trial by Alhazmi et al., who compared both brief relaxation therapy and auditory distraction for the reduction of dental anxiety in patients undergoing tooth extraction. Although both techniques proved effective, the study found brief relaxation therapy to be superior to the auditory distraction technique. However, auditory distraction was a more applicable and manageable option for reducing dental anxiety in the current study setting, where cost‐effectiveness and timely completion of the protocol were critical due to substantial outpatient department turnover in the OMFS department and a shortage of clinicians. Brief relaxation therapy, on the other hand, requires trained personnel like physiotherapists and cannot be implemented without training, hence it was not prioritized.

The technique used in this study first established baseline anxiety levels in the waiting room and then measured them again just before extraction. While numerous previous studies have assessed anxiety scores post‐extraction rather than pre‐extraction, the researchers believe this approach produces subjective results; once the dental procedure is complete, anxiety levels understandably decrease (Gazal et al., 2016; Lahmann et al., 2008). Therefore, the current study assessed the effectiveness of the anxiety reduction protocol pretreatment rather than posttreatment.

The findings of this study are also supported by a systematic review by Hoffman et al. (2022), which assessed anxiety by measuring physiological parameters and concluded that music aids in distracting the patient and also suppresses the autonomic nervous system.

It is noteworthy that in the current study, the researchers allowed patients to use headphones and choose their preferred music. This approach mirrors Saravanan's demonstration that using headphones to listen to music during dental treatment allows patients to control the volume and mask surrounding noises from drilling (Wong et al., 2018). These findings highlight the superior efficacy of headphones in alleviating dental anxiety. Additionally, the current study allowed patients to choose their own music, reaffirming the findings of another study that, irrespective of the type, listening to music significantly reduces dental anxiety (Gulnahar & Kupeli, 2020).

Numerous studies have established auditory distraction as an effective tool for reducing dental anxiety. Some studies have tested the effectiveness of both audio and audio‐visual distraction aids for managing pain and anxiety, while others assessed their effectiveness in specific clinical procedures like root canal treatment (Gurav et al., 2022; Jethani et al., 2019). The study firmly endorses the use of auditory distraction as an effective approach for reducing dental anxiety in patients undergoing nonsurgical extraction. It demonstrates the potential for this noninvasive, patient‐centered intervention to significantly decrease anxiety levels before dental procedures, thereby improving the overall patient experience. Prior studies have also suggested that when participants listened to music it proved to be an effective tool in reducing their physical and mental stress, as well as increasing their safety and pain inception (Hilliard, 2005).

Auditory distraction, as implemented in this study, presents several notable advantages. It is a cost‐effective solution, especially pertinent in resource‐limited settings like Pakistan. Additionally, it can be easily integrated into existing dental care protocols without the need for specialized training, further emphasizing its practicability.

The insignificant impact of demographic and clinical characteristics on the outcomes of our study supports the broad applicability of auditory distraction across diverse patient populations, thus showing promise in its potential to universally improve dental care experiences.

While our findings are promising, further research involving a larger sample size is essential to comprehensively evaluate the efficacy and constraints of auditory distraction across diverse dental procedures. Additionally, investigating other noninvasive techniques for managing dental anxiety is recommended to broaden the array of tools available for anxiety mitigation in dental practices. Our study, however, is not without limitations. First, the small sample size may restrict the generalizability of our findings beyond the immediate study context. Second, the results do not support causal interpretations or implications.

## 5 CONCLUSION

Adoption of patient‐centered interventions such as auditory distraction can significantly contribute to reducing dental anxiety, thereby potentially improving oral health outcomes and overall patient satisfaction and compliance in dental care.

## AUTHOR CONTRIBUTIONS

Sheze Haroon Qazi and Saba Masoud conceptualized the research idea and prepared the initial draft of the article. Khadija Tariq and Minahil Khan were responsible for conducting the study under real clinical situations, under the guidance and supervision of Sheze Haroon Qazi and Saba Masoud. Reza Emrani played a crucial role in proofreading and reviewing the manuscript, contributing significantly to its finalization. Javed Ashraf was responsible for the visulaizations, statisitcal anlayses, finalization and submission of the manuscript. Additionally, Javed Ashraf supervised, managed and finalized the manuscript revisions and handled the resubmission process. Sheze Haroon Qazi and Saba Masoud reviewed and proofread the revised version of the manuscript, ensuring its readiness for publication.

## CONFLICT OF INTEREST STATEMENT

The authors declare no conflict of interest.

## Data Availability

The data that support the findings of this study are available from the corresponding author upon reasonable request.
